# Prognostic Value of Residual Disease after Interval Debulking Surgery for FIGO Stage IIIC and IV Epithelial Ovarian Cancer

**DOI:** 10.1155/2015/464123

**Published:** 2015-05-27

**Authors:** Marianne J. Rutten, Gabe S. Sonke, Anneke M. Westermann, Willemien J. van Driel, Johannes W. Trum, Gemma G. Kenter, Marrije R. Buist

**Affiliations:** ^1^Centre for Gynaecologic Oncology Amsterdam, Academic Medical Centre, Amsterdam, Netherlands; ^2^Department of Medical Oncology, Netherlands Cancer Institute, P.O. Box 22700, 1100 DE Amsterdam, Netherlands; ^3^Department of Medical Oncology, Academic Medical Centre, Amsterdam, Netherlands; ^4^Centre for Gynaecologic Oncology Amsterdam, Netherlands Cancer Institute, Netherlands; ^5^Centre for Gynaecologic Oncology Amsterdam, Free University Medical Centre, Amsterdam, Netherlands

## Abstract

Although complete debulking surgery for epithelial ovarian cancer (EOC) is more often achieved with interval debulking surgery (IDS) following neoadjuvant chemotherapy (NACT), randomized evidence shows no long-term survival benefit compared to complete primary debulking surgery (PDS). We performed an observational cohort study of patients treated with debulking surgery for advanced EOC to evaluate the prognostic value of residual disease after debulking surgery. All patients treated between 1998 and 2010 in three Dutch referral gynaecological oncology centres were included. The prognostic value of residual disease after surgery for disease specific survival was assessed using Cox-regression analyses. In total, 462 patients underwent NACT-IDS and 227 PDS. Macroscopic residual disease after debulking surgery was an independent prognostic factor for survival in both treatment modalities. Yet, residual tumour less than one centimetre at IDS was associated with a survival benefit of five months compared to leaving residual tumour more than one centimetre, whereas this benefit was not seen after PDS. Leaving residual tumour at IDS is a poor prognostic sign as it is after PDS. The specific prognostic value of residual tumour seems to depend on the clinical setting, as minimal instead of gross residual tumour is associated with improved survival after IDS, but not after PDS.

## 1. Introduction

Advanced epithelial ovarian cancer (EOC) is the leading cause of gynaecologic cancer death. It is treated with a combination of cytoreductive surgery and chemotherapy. Despite advances in chemotherapeutic agents and more radical surgery, there has been little improvement in overall survival in the past decades [1]. A randomised trial showed similar survival rates between patients with advanced stage EOC treated with neoadjuvant chemotherapy and interval debulking surgery (NACT-IDS) or primary debulking surgery (PDS) and adjuvant chemotherapy [2]. However, complete resection of all macroscopic tumour at debulking surgery showed to be the single most important independent prognostic factor in advanced ovarian carcinoma [2–6]. The primary objective of debulking surgery in ovarian cancer is complete removal of all visible disease [3, 7]. As the result of surgery is a very important and modifiable prognostic factor, no macroscopic residual disease should be pursued to obtain the best prognosis [3, 7–10]. Although this is more often achieved at IDS, it does not result in appreciably better overall survival. Thus, the prognostic value of tumour residual at debulking surgery appears different in patients who received NACT compared to PDS [3, 11, 12].

In this study we evaluated the prognostic significance of residual disease after primary debulking surgery and after interval debulking surgery.

## 2. Methods

### 2.1. Patients

Consecutive patients who underwent cytoreductive surgery at one of three oncologic centres in the north western part of Netherlands (Academic Medical Centre, Free University Medical Centre, and Netherlands Cancer Institute) for primary epithelial ovarian, fallopian tube, or peritoneal cancer (EOC) FIGO stage IIIC/IV between January 1998 and August 2010 were identified from a prospective clinical cancer registry.

All patients underwent surgery by gynaecologic oncologists. Patients referred from a nononcologic centre after prior suboptimal surgery by a general gynaecologist were excluded. Staging of disease was done according to FIGO (2006) criteria for ovarian carcinoma. Every operative cytoreductive procedure was performed with the aim of leaving NRD. PDS was performed if in the opinion of the multidisciplinary team, consisting of gynaecologic oncologists, medical oncologists, and a dedicated radiologist, debulking surgery of all visible tumour to less than one centimetre in diameter was possible. Patients with more extensive disease and those unable to undergo surgery started neoadjuvant chemotherapy. Patients who underwent exploratory laparotomy for diagnostic biopsy or oophorectomy without debulking were analysed in the IDS group.

Results of surgery were qualified as no residual disease (NRD), minimal residual disease (MRD; deposits of residual tumour <1 cm), or gross residual disease (GRD; deposits of residual tumour >1 cm).

Standard procedures at PDS as well as at IDS included midline laparotomy, hysterectomy, bilateral salpingooophorectomy, infragastric omentectomy, and removal of all macroscopic tumour if possible. Surgery was classified as extensive if additional interventions such as diaphragmatic and peritoneal stripping, (partial) liver resection, splenectomy, bowel resection, or pelvic- and para-aortic lymphadenectomy were performed to achieve at least MRD. This was performed when it was thought to aid in cytoreductive outcome to at least MRD. Patient data were abstracted from the clinical cancer registry. Information included demographic data, laboratory results, surgical findings, interventions at surgery and results, pathology, treatment, and follow-up data.

### 2.2. Analysis

Treatment characteristics were compared using chi-square and Student's *t*-test or Mann-Whitney *U* test when appropriate. Progression-free survival and disease specific survival were calculated from the date of first surgery, or start of chemotherapy in case of NACT, to the documented date of progression, respectively, death or last follow-up, whichever occurred first. Impact of surgery result on survival was assessed by constructing Kaplan-Meier curves with a log-rank test. Cox-regression analyses were performed to assess the influence of residual disease in combination with other prognostic factors on survival. All reported significance was 2-tailed at a level of 0.05. Statistical analysis was performed using SPSS statistical software, version 20.

## 3. Results

In the study period 689 patients were surgically treated for primary EOC FIGO stage IIIC or stage IV. The characteristics of the patients are shown in Table 1. Mean age of all patients was 62 years. The majority of patients had FIGO stage IIIC disease, serous histology, and grade 3 tumour. Median follow-up was 62 months (range 0.9–165 months).

In total, 462 patients were treated with NACT and IDS. The remaining 227 patients were treated with primary debulking surgery. Within the group of patients treated with NACT-IDS, 134 underwent an explorative laparotomy or laparoscopy before start of chemotherapy to assess the operability and to obtain histology for diagnosis, but without debulking surgery.

There were 41 patients in the PDS group who underwent IDS after 2-3 courses of chemotherapy because of GRD after PDS. No difference in survival was observed between patients with GRD after PDS who subsequently had IDS compared to those who did not. Therefore patients who underwent PDS as well as IDS were analysed in the PDS group. Median disease specific survival of the total population was 35 months.

Of all patients, 254 had extensive surgery. This percentage did not differ between patients treated with PDS or IDS. NRD was achieved at debulking surgery in 36% and 46% of patients in the PDS and IDS group, respectively (Table 2). At IDS this was more often achieved without extensive surgery.

Chemotherapy mostly was administered as a carboplatin/paclitaxel combination, although single agent carboplatin and other combinations were sometimes administered (Table 1). In both treatment groups patients received a median of six cycles of chemotherapy (range 0–13). Before IDS patients received 3 (range 1–10) cycles of chemotherapy.

### 3.1. Prognostic Factors for Survival after PDS

**Figure 1:**
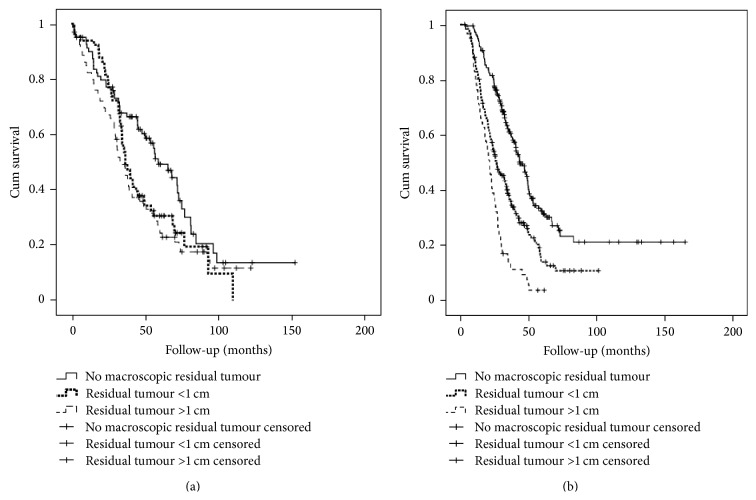
Disease specific survival according to surgery result for (a) primary debulking surgery (PDS) and (b) interval debulking surgery (IDS).

Median follow-up for patients treated with PDS was 74 months (range 1–152). Mortality within 30 days after surgery was less than one percent. Progression within six months after the last cycle of chemotherapy was seen in 32% of patients treated with PDS (Table 3). Median progression-free survival (PFS) was 17 months and median disease specific survival (DSS) 40 months (Figure 1).

Completeness of surgery was an important prognostic factor. DSS with NRD was 57 months compared with 36 months after both MRD and GRD (HR MRD versus NRD 1.5 (95% CI 1.0–2.2), HR GRD versus NRD 1.6 (95% CI 1.1–2.4)). MRD did not result in prolonged survival compared to GRD. The adjusted HR for MRD versus GRD was HR 0.9 (95% CI 0.6–1.3). In multivariable analysis residual disease was an independent prognostic factor. The corresponding HRs were 2.0 (95% CI 1.1–3.8) and 1.8 (95% CI 1.1–3.2). Other independent predictors for DSS were performance status (HR 2.0 (95% CI 1.3–3.1)) and mucinous or clear cell histology versus others (HR 2.9 95% CI 1.4–6.2 and 2.7; 95% CI 1.3–5.6 resp.). Extensive surgery did not result in prolonged survival (HR 0.8 (95% CI 0.6–1.3)). The results of the univariable and multivariable analysis are presented in Table 4(a).

### 3.2. Prognostic Factors for Survival after IDS

Median follow-up for patients with IDS was 55 months (range 3–165). Mortality within 30 days after surgery was less than one percent. Progression within six months after the last cycle of chemotherapy was seen in 40% of patients after IDS (Table 2). Median progression-free survival (PFS) was 14 months and median disease specific survival (DSS) 33 months (Figure 1).

Completeness of surgery was the most important prognostic factor. DSS with NRD was 44 months compared with 27 months with MRD and 22 months with GRD (HR MRD versus NRD 1.9 (95% CI 1.5–2.4), HR GRD versus NRD 3.4 (95% CI 2.5–4.7)). The results of the univariable and multivariable analysis are presented in Table 4(b). In multivariable analyses residual disease was the only independent prognostic factor. The corresponding HRs were 1.8 (95% CI 1.3–2.5) and 3.1 (95% CI 2.0–4.8). The adjusted HR for MRD versus GRD was 0.6 (95% CI 0.4–0.8). Although at univariable analysis large volume ascites predicted worse prognosis, it was not an independent predictor. Extensive surgery at interval debulking surgery did not result in better survival (HR 1.1 (95% CI 0.9–1.4)).

## 4. Discussion

To our knowledge this is the largest cohort analysing prognostic factors in patients treated with NACT-IDS for EOC outside the realm of a clinical trial. Absence of residual disease after debulking surgery was confirmed to be a strong prognostic marker for disease specific survival after IDS as it is after PDS. Yet, the prognostic value of residual disease depends on the clinical setting. Patients selected for PDS left without residual disease after debulking surgery have the longest survival. Even so, NRD at IDS results in longer survival than leaving any residual disease. Furthermore, achievement of minimal rather than gross residual disease at IDS results in significantly prolonged survival, whereas this effect was not confirmed for PDS.

In accordance with other studies, NRD was achieved more often in the IDS group than in the PDS group, although this did not confer a survival benefit [12, 13]. A likely explanation for this discrepancy in our cohort study is the selection of patients for NACT-IDS based on tumour load and comorbidity. Another possible explanation is the induction of fibrosis with NACT, which might masquerade tumour deposits and thus lead to an overestimation of the completeness of surgery [14–16]. This hypothesis is supported by the findings of Hynninen et al. [11], who recently reported a lower sensitivity for identifying malignant sites after NACT than at primary debulking surgery [9]. Finally, the development of platinum resistance during NACT has been suggested by exposing larger tumour volumes to chemotherapy [17, 18].

Chang et al. [19] previously stated that radical surgery leads to better overall survival when patients are treated with PDS. We could not confirm this in our PDS group, which could be due to the size of our population. Yet, patients with extensive disease diagnosed on computed tomography imaging or at diagnostic surgery were not randomly selected for NACT and no effort to perform extensive primary surgery was made in this group, resulting in selection bias.

The long and near-complete follow-up with known cause of death for all deceased patients is a strength of this study. However, patients were not followed from first date of visiting the Outpatient Department, but from start of treatment, respectively, first surgery, either diagnostic or therapeutic, or start of NACT. We have chosen this moment of start of follow-up because a number of patients were referred to our centres for treatment and their first date of contact in hospitals elsewhere was not known. Yet, since moment of start of the treatment strategies was calculated in the same way, it is unlikely that bias is introduced within the treatment groups regarding survival.

As in any observational study, formal comparison of outcome between treatment groups is hampered by confounding by indication and therefore invalid. PDS was performed if, in the opinion of the multidisciplinary team, debulking surgery of all visible tumour to less than one centimetre in diameter was possible. Patients with more extensive disease and patients unfit to undergo surgery started neoadjuvant chemotherapy. Therefore, in our series, patients receiving NACT had more extensive disease and generally worse performance status. The value of prognostic markers such as the degree of residual disease after surgery, however, can be compared. We showed that patients who have no residual disease after NACT-IDS or PDS and adjuvant chemotherapy have the best prognosis. This is consistent with published studies. Moreover, if at PDS it is not feasible to achieve NRD there is a higher chance to obtain this at IDS, and this will improve prognosis [2, 12, 20].

If at IDS NRD cannot be achieved, all possible effort, including extensive surgery, should be performed to achieve at least residual disease of less than one centimeter. Current diagnostic work-up is not adequate and new diagnostic tools are needed to optimize selection of patients for primary surgery or NACT [21]. Laparoscopy is currently studied in a randomised trial of patient selection [6].

In conclusion, NRD should be the goal of all cytoreductive surgery in ovarian cancer. Therefore selection of patients for treatment is of utmost importance.

## Figures and Tables

**Table 1:** Baseline characteristics.

	All patients (*n* = 689)	PDS (*n* = 227)	IDS (*n* = 462)
Age; mean (SD)	61.5 (10.7)	59.4 (31–86)	62.5 (29–83)
WHO performance status, number (%)			
0	310 (45.0)	122 (53.7)	188 (40.7)
1	218 (31.6)	63 (27.7)	155 (33.5)
2	53 (7.7)	13 (5.6)	40 (8.7)
3	9 (1.3)	2 (0.1)	7 (1.5)
Missing	99 (14.4)	27 (11.9)	72 (15.6)
ASA-score, number (%)			
1	198 (28.7)	62 (27.4)	136 (29.4)
2	370 (53.7)	119 (52.4)	251 (54.2)
3	88 (12.8)	35 (15.4)	53 (11.5)
Missing	34 (4.9)	11 (4.8)	23 (4.9)
FIGO stage, number (%)			
IIIC	543 (78.8)	209 (92.1)	334 (72.3)
IV	146 (21.2)	18 (7.9)	128 (27.7)
Histologic type, number (%)			
Serous	502 (72.9)	156 (68.7)	346 (74.8)
Mucinous	24 (3.5)	12 (5.3)	12 (2.6)
Endometrioid	47 (6.8)	29 (12.8)	18 (3.9)
Clear cell	20 (2.9)	11 (4.8)	9 (2.0)
Undifferentiated	86 (12.5)	12 (5.3)	74 (16.1)
Mixed/other	10 (1.5)	7 (3.0)	3 (0.6)
Histologic grade, number (%)			
Well differentiated	32 (4.6)	15 (6.6)	17 (3.7)
Moderately differentiated	103 (14.9)	47 (20.7)	56 (12.1)
Poorly differentiated	373 (54.1)	141 (62.1)	232 (50.2)
Missing	181 (26.3)	24 (10.6)	157 (34.0)
CA 125 before treatment; median (range)	908.0 (12–67448)	807.5 (12–67448)	1041 (15–42077)
Ascites at surgery (ml); median (range)	500 (0–70000)	200 (0–12000)	500 (0–70000)
Cycles of chemotherapy	6 (0–13)	6 (0–9)	6 (0–13)
Type of chemotherapy			
Carboplatin/paclitaxel	641 (93)	197 (87)	444 (96)
Multidrug without platinum	10 (1.5)	3 (1)	7 (2)
Single drug platinum	27 (3.9)	17 (8)	10 (2)
No chemotherapy received	8 (1.2)	8 (4)	0 (0.0)
Missing	3 (0.4)	2 (1)	1 (0)

**Table 2:** Treatment results according to treatment group. Values given are numbers (%).

	PDS patients (*n* = 227)	IDS patients (*n* = 462)
Residual disease		
No macroscopic tumour	81 (36)	213 (46)
Minimal residual (<1 cm)	67 (30)	187 (41)
Gross residual (>1 cm)	79 (35)	60 (13)
Missing	0 (0)	2 (0)
Extensive surgery	85 (39)	169 (38)
Missing	9 (4)	19 (4)

**Table 3:** Survival outcome according to timing of surgical treatment. Values given are numbers (%) or months (SE) for DSS and PFS.

Survival	PDS patients (*n* = 227)	IDS patients (*n* = 462)
Mortality < 30 days after surgery (%)	2 (1)	2 (0)
DSS in months		
NRD	59.2 (7)	43.6 (3)
MRD	36.7 (3)	26.7 (3)
GRD	35.6 (4)	21.5 (2)
PFS in months		
NRD	23.7 (4)	17.4 (1)
MRD	16.7 (2)	11.9 (1)
GRD	12.5 (1)	9.0 (1)
Progression within 6 months after last cycle of chemotherapy	68 (32)	182 (40)

DSS: disease specific survival; PFS: progression-free survival; NRD: no residual disease; MRD: minimal residual disease (deposits of residual tumour <1 cm); GRD: gross residual disease (deposits of residual tumour >1 cm).

**(a):** 

	Univariable analysis			Multivariable analysis		
	HR	95% CI	*p* value	HR	95% CI	*p* value
Age (years)	1.00	0.99–1.02	0.55	1.01	0.99–1.03	0.47
FIGO stage IV	1.46	0.82–2.58	0.20	1.06	0.45–2.47	0.90
WHO performance ≥ 1	2.02	1.44–2.85	<0.001	**2.01**	**1.32–3.07**	**<0.001**
ASA ≥ 2	1.10	0.78–1.56	0.59	0.80	0.38–0.80	0.38
Histology						
HGS and undifferentiated	1			1		
Low grade serous	0.39	0.10–1.58	0.19	0.19	0.03–1.45	0.11
Mucinous	1.93	1.04–3.60	0.04	**2.93**	**1.38–6.20**	**0.01**
Endometrioid	0.83	0.50–1.36	0.46	0.81	0.41–1.62	0.56
Clear cell	1.81	0.95–3.48	0.07	**2.69**	**1.29–5.62**	**0.01**
CA 125 before treatment	1.07	0.98–1.18	0.15	0.97	0.84–1.11	0.63
Intraoperative ascites > 500 ml	1.35	0.97–1.06	0.08	0.89	0.56–1.41	0.61
Extensive surgery	0.85	0.61–1.18	0.34	0.84	0.55–1.27	0.41
Residual disease						
NRD	1			1		
MRD	1.48	0.99–2.21	0.05	**2.04**	**1.11–3.76**	**0.02**
GRD	1.64	1.13–2.39	0.01	**1.84**	**1.05–3.21**	**0.03**

**(b):** 

	Univariable analysis			Multivariable analysis		
	HR	95% CI	*p* value	HR	95% CI	*p* value
Age (years)	**1.01**	**1.00–1.02**	**0.03**	1.02	0.99–1.03	0.06
FIGO stage IV	1.09	0.85–1.38	0.51	1.17	0.80–1.71	0.41
WHO performance ≥ 1	**1.32**	**1.03–1.68**	**0.03**	0.96	0.70–1.32	0.80
ASA ≥ 2	1.19	0.93–1.53	0.16	1.06	0.75–1.49	0.75
Histology						
HGS and undifferentiated	1			1		
Low grade serous	0.66	0.29–1.49	0.32	0.34	0.11–1.08	0.08
Mucinous	1.49	0.74–3.03	0.27	2.05	0.74–5.73	0.17
Endometrioid	0.77	0.41–1.46	0.43	1.24	0.64–2.40	0.53
Clear cell	1.08	0.51–2.31	0.83	1.48	0.60–3.71	0.40
CA 125 before treatment				0.94	0.84–1.05	0.25
Intraoperative ascites > 500 ml	**1.68**	**1.26–2.24**	**<0.001**	1.41	0.93–2.15	0.10
Extensive surgery	0.92	0.73–1.16	0.49	1.09	0.86–1.37	0.08
Residual disease						<0.001
NRD	1			1		
MRD	**1.87**	**1.47–2.39**	**<0.001**	**1.79**	**1.26–2.53**	**<0.001**
GRD	**3.42**	**2.48–4.72**	**<0.001**	**3.11**	**2.01–4.81**	**<0.001**

HR: hazard ratio, CI: confidence interval, HGS: high grade serous, NRD: no residual disease, MRD: minimal residual disease (deposits of residual tumour <1 cm), and GRD: gross residual disease (deposits of residual tumour >1 cm).
